# HFSOF: A Hierarchical Feature Selection and Optimization Framework for Ultrasound-Based Diagnosis of Endometrial Lesions

**DOI:** 10.3390/biomimetics11010074

**Published:** 2026-01-15

**Authors:** Yongjun Liu, Zihao Zhang, Tongyu Chai, Haitong Zhao

**Affiliations:** 1 School of Computer Science and Engineering, Suzhou University of Technology, Suzhou 215500, China; z09223101@szut.edu.cn; 2 School of Computer Science and Engineering, Shenyang Jianzhu University, Shenyang 110168, China; aebestach@stu.sjzu.edu.cn

**Keywords:** endometrial lesions, machine learning, feature engineering, swarm intelligence, SVM

## Abstract

Endometrial lesions are common in gynecology, exhibiting considerable clinical heterogeneity across different subtypes. Although ultrasound imaging is the preferred diagnostic modality due to its noninvasive, accessible, and cost-effective nature, its diagnostic performance remains highly operator-dependent, leading to subjectivity and inconsistent results. To address these limitations, this study proposes a hierarchical feature selection and optimization framework for endometrial lesions, aiming to enhance the objectivity and robustness of ultrasound-based diagnosis. Firstly, Kernel Principal Component Analysis (KPCA) is employed for nonlinear dimensionality reduction, retaining the top 1000 principal components. Secondly, an ensemble of three filter-based methods—information gain, chi-square test, and symmetrical uncertainty—is integrated to rank and fuse features, followed by thresholding with Maximum Scatter Difference Linear Discriminant Analysis (MSDLDA) for preliminary feature selection. Finally, the Whale Migration Algorithm (WMA) is applied to population-based feature optimization and classifier training under the constraints of a Support Vector Machine (SVM) and a macro-averaged F1 score. Experimental results demonstrate that the proposed closed-loop pipeline of “kernel reduction—filter fusion—threshold pruning—intelligent optimization—robust classification” effectively balances nonlinear structure preservation, feature redundancy control, and model generalization, providing an interpretable, reproducible, and efficient solution for intelligent diagnosis in small- to medium-scale medical imaging datasets.

## 1. Introduction

Uterine endometrial states, encompassing normal endometrium, polyps, hyperplasia, fibroids, endometrial carcinoma, and other pathological changes, are common in gynecological practice and exhibit considerable clinical heterogeneity. These conditions can significantly impact menstrual function, fertility, and overall reproductive health, with certain lesions, such as fibroids and endometrial carcinoma, carrying potential for substantial morbidity if not accurately identified and managed [1,2,3]. Imaging plays a pivotal role in the diagnosis and characterization of these endometrial states, with ultrasonography recognized as the first-line, non-invasive modality due to its accessibility, low cost, and real-time evaluation capabilities [4,5]. However, sonographic assessment is inherently operator-dependent, and diagnostic accuracy can be compromised by subjectivity and interobserver variability. Consequently, the development of machine learning-based approaches, including feature selection and classification algorithms, offers a promising strategy to objectively differentiate normal endometrium from various pathological states, thereby supporting more consistent and precise clinical decision-making.

In recent years, deep learning (DL) has shown considerable potential in medical image analysis, including lesion detection and classification tasks across diverse modalities. Numerous studies have demonstrated high performance in tasks such as breast cancer detection in mammography or urothelial carcinoma identification in cytology; however, reproducibility and interpretability remain major concerns [6,7,8]. Many DL approaches are sensitive to limited sample sizes, heterogeneous imaging protocols, and substantial variability in lesion appearance, which often leads to unstable training, overfitting, and reduced generalization in clinical practice. Moreover, most models operate as end-to-end black boxes, offering little insight into the contribution of specific features to diagnostic decisions. Feature selection and optimization have been proposed as a complementary strategy to address these limitations, thereby enabling dimensionality reduction, improving model interpretability, and enhancing robustness, particularly for small- to medium-scale datasets. Therefore, developing an efficient, reproducible, and interpretable feature engineering pipeline, coupled with a robust classifier, represents a practical approach to improving diagnostic performance under realistic constraints of clinical imaging data.

Although classification and diagnosis of endometrial diseases are clinically important, most studies focus on molecular or genomic analyses, while imaging-based classification remains limited [9,10,11,12]. Existing attempts to combine imaging features with machine learning often rely on small sample sizes, are limited to specific menstrual phases, and lack systematic feature selection or classification strategies. Consequently, the potential of imaging for disease subtyping, risk assessment, and personalized management remains underexplored. Developing approaches that integrate imaging feature engineering with robust machine learning classifiers could enable accurate classification with limited samples, improve model interpretability and reproducibility, and offer a novel path for precise diagnosis of endometrial diseases.

Inspired by studies [13,14] on machine learning-based classification frameworks, we recognized several opportunities to extend prior approaches for ultrasound imaging of the endometrium. Notably, our previous work [14] developed a BPSO-based feature selection framework for endometrium detection, demonstrating the effectiveness of machine learning in ultrasound image analysis. Building upon that foundation, the present study expands the scope from single-category detection to multi-class classification of endometrial states, aiming to capture more comprehensive pathological information. As endometrial ultrasound images often contain substantial noise and exhibit highly nonlinear characteristics, traditional linear methods are limited in their ability to represent intrinsic structures. To address this, we adopt KPCA [15,16] to extract non-linear feature representations while reducing dimensionality. The KPCA-mapped features effectively reveal complex imaging patterns but may still contain redundancy. Therefore, a subsequent filter-based feature selection method is employed to retain the most discriminative features, enhancing classifier robustness and mitigating noise and overfitting.

To further refine the feature set, we employ MSDLDA [17], which maximizes the difference between between-class and within-class scatter, providing a stable and reliable criterion for preliminary feature selection. However, MSDLDA primarily evaluates features based on class scatter statistics and does not account for interactions between features or the overall performance of the classifier. Therefore, a subsequent global optimization is necessary to identify the most informative subset of features that maximizes classification performance. To this end, we adopt a population-based optimization strategy using the WMA [18], which efficiently explores the global search space and selects feature subsets under macro-average F1 score constraints.

Based on these considerations, we propose a hierarchical feature selection and optimization framework for six endometrial categories. The framework follows a closed-loop design, in which non-linear kernel-based dimensionality reduction is first performed, followed by ensemble filter-based feature ranking. Preliminary feature selection is then conducted using MSDLDA, and subsequent population-based optimization is carried out using WMA. Finally, a classifier is trained on the optimized feature set. This pipeline balances non-linear structure preservation, redundancy control, and global search optimization while maintaining model generalizability, making it well-suited for real-world ultrasound datasets with heterogeneous imaging conditions.

The contributions of the research lie in the following:

(1) We introduce a non-linear kernel-based dimensionality reduction strategy to capture the intrinsic structure of ultrasound imaging features, enabling more effective representation of endometrial characteristics.
(2) Ensemble filter-based feature ranking combined with MSDLDA is employed for preliminary feature selection, providing a stable and reliable criterion to reduce feature redundancy and enhance discriminative power.
(3) A population-based optimization approach using WMA is applied to select the most informative feature subset, maximizing classification performance under macro-average F1 score constraints and capturing feature interactions.

The remainder of the paper follows this format: Section 2 reviews related work, covering traditional image features with machine learning, deep learning approaches, nonlinear dimensionality reduction and kernel methods, feature selection and fusion, and metaheuristic optimization for feature selection. Section 3 defines the problem under study. Section 4 presents the proposed methodology, including nonlinear dimensionality reduction using kernel principal component analysis, ensemble filter-based feature selection with median fusion, linear discriminant analysis-based thresholding for feature refinement, and wrapper optimization via the WMA algorithm. Section 5 describes the experimental setup, datasets, results, and comparative analyses. Section 6 ends the study by explaining future work.

## 2. Related Work

### 2.1. Traditional Radiomic Features and Machine Learning Approaches

Traditional radiomic analysis [19,20], characterized by handcrafted feature extraction and classical machine learning (ML) modeling, had long served as an essential approach for medical image quantification and disease classification. Texture descriptors such as gray-level co-occurrence matrix (GLCM)-derived Haralick features [21,22] and wavelet-based [23] statistics were widely applied to capture intratumoral heterogeneity and tissue microstructure. When coupled with classifiers such as SVM, random forests (RF), and k-nearest neighbors (KNN), these features demonstrated reliable performance, particularly in small- to medium-sized datasets where interpretability and stability were critical.

For instance, Li et al. [24] proposed an artificial intelligence-enhanced ultrasound analysis system for endometrial cancer screening that combined radiomics features extracted via Pyradiomics with convolutional neural network–derived representations. The resulting hybrid deep learning–radiomics model achieved the highest diagnostic performance (AUROC = 0.893), outperforming both single-modality approaches and effectively reducing observer variability through super-resolution-based preprocessing. Similarly, Xu et al. [25] utilized ultrasound-based radiomics to predict reproductive outcomes following frozen embryo transfer (FET). By integrating the radiomics score and clinical variables using the XGBoost algorithm, the fusion model achieved an AUC of 0.861, significantly outperforming models based on either feature set alone.

These examples demonstrated that traditional handcrafted radiomic features, when combined with robust ML algorithms and clinical context, could yield accurate and interpretable models. Classical machine learning, therefore, remained a practical and explainable foundation for quantitative medical imaging, motivating the use of ML-based methods in this study.

### 2.2. Deep Learning-Based Methods

Recent advances in deep learning have greatly enhanced medical image analysis by enabling automated disease diagnosis and lesion characterization. However, challenges such as limited annotations, data imbalance, and inter-device variability continued to hinder model generalization, particularly in ultrasound imaging.

For example, Jiang et al. [26] introduced SGLA-Net, a segmentation knowledge-based global-local attention classification network for breast tumor diagnosis in ultrasound images. The Global–Local Feature Interaction (GLFI) block allowed adaptive fusion of fine-grained local and global features, achieving superior segmentation Dice coefficients and classification AUC compared with state-of-the-art baselines. Similarly, Cai et al. [27] developed the Dynamic Adaptive Fusion Network (DAFNet), a lightweight and efficient architecture that employed dynamic attention and adaptive normalization for cross-domain medical image classification. DAFNet outperformed common backbones such as ResNet and EfficientNet while maintaining lower computational costs.

Although deep learning approaches achieved impressive performance, they often suffered from high data dependence, instability during training, and limited interpretability. These constraints highlighted the importance of maintaining machine learning-based frameworks that balanced accuracy, transparency, and robustness in medical imaging research.

### 2.3. Dimensionality Reduction and Nonlinear Feature Embedding

High-dimensional radiomic and textural descriptors extracted from medical images often suffer from the curse of dimensionality, leading to feature redundancy and model overfitting. Classical linear dimensionality reduction methods, such as Principal Component Analysis (PCA) and Linear Discriminant Analysis, have been widely used to address these issues by projecting data into compact, discriminative subspaces. These approaches enhanced computational efficiency and improved model generalization; however, their inherent linear assumptions often failed to capture the complex and nonlinear manifold structures present in medical imaging data, particularly in ultrasound modalities characterized by intricate speckle textures and heterogeneous gray-level distributions.

Recent studies explored both linear and nonlinear dimensionality reduction in biomedical contexts. Azmal et al. [28] applied PCA in a multi-omics deep learning framework to reduce dimensionality while retaining biologically meaningful variance. Hayum et al. [29,30] employed KPCA after extracting textural and morphological features, followed by bio-inspired feature selection, achieving improved classification accuracy in breast cancer datasets. Mohammed combined logistic regression with KPCA and LS-SVM, yielding higher diagnostic accuracy than conventional classifiers. Fazal et al. [31] integrated KPCA with sparse-representation classification for anticancer peptide prediction, enhancing feature discrimination and classification balance. Avian et al. [32] incorporated KPCA into E-nose signal processing for COPD and lung cancer detection, improving class separability and model consistency.

Despite the extensive exploration of kernel-based dimensionality reduction in diverse imaging and omics applications, a thorough literature survey revealed that no prior study had employed such nonlinear embedding methods for conventional B-mode ultrasound imaging of the endometrium. To address this gap, the present study employed KPCA to perform effective nonlinear dimensionality reduction, aiming to capture the underlying manifold structure of endometrial echogenicity while preserving diagnostically relevant discriminative features. Compared with linear projection methods, KPCA was particularly advantageous for ultrasound-based analysis, as endometrial ultrasound features often exhibited strong nonlinear relationships, complex scattering effects, and considerable speckle noise. These characteristics weakened linear correlations and made them difficult to extract, whereas KPCA mapped the data into a high-dimensional kernel space where nonlinear variations became linearly separable. This property enabled KPCA to better preserve subtle but diagnostically important structural patterns, enhance feature discriminability, and improve robustness against noise inherent in B-mode imaging.

### 2.4. Feature Selection and Fusion

Feature selection and fusion played a crucial role in improving classification robustness and model interpretability, especially in high-dimensional biomedical imaging and multi-omics data. Integrating multi-level features and hybrid selection strategies effectively enhanced diagnostic performance while reducing redundancy.

Zheng et al. [33] combined radiomic descriptors with deep learning features from multiple CNNs (AlexNet, GoogLeNet, VGG16, ResNet50) and applied T-test filtering, LASSO, and PCA. The fused features outperformed single-source representations (97.6% vs. 90.3%), highlighting the benefit of complementary feature integration.

Li et al. [34] applied a Pareto-guided recursive feature selection on high-dimensional multi-omics data, identifying 76 key biomarkers and achieving 95.3% accuracy. This study demonstrated how optimization-driven selection balanced discriminability and interpretability.

Krishana et al. [35] evaluated different feature selection methods on clinical and laboratory data for PCOS diagnosis. Backward elimination combined with Random Forest achieved 96.55% accuracy, highlighting key discriminative features such as AMH levels and endometrium thickness.

These studies illustrated the value of adaptive, hybrid feature engineering across imaging, omics, and clinical data. In the present study, the proposed feature selection strategy further enhanced model performance by efficiently pruning redundant or noisy descriptors, producing a compact yet highly discriminative feature set that improved both predictive accuracy and interpretability.

### 2.5. Meta-Heuristic Optimization for Feature Selection

The integration of meta-heuristic optimization techniques into medical image analysis has demonstrated strong potential to improve diagnostic accuracy while managing high-dimensional features. In endometrial cancer, Brar et al. [36] applied an Extra Tree–Whale Optimization Feature Selector (ET-WOFS) on heterogeneous shallow and deep features, achieving classification accuracy exceeding 95% with low false positive and negative rates.

In liver cirrhosis classification, Karthikamani et al. [37] combined Particle Swarm Optimization (PSO) with hybrid approaches (AAO-GMM) to optimize features from Fuzzy C-Means and Possibilistic FCM, reaching accuracies above 99%.

Algubili et al. [38] enhanced the Grey Wolf Optimizer with a dynamic fuzzy system (FGWO) to maintain population diversity and reduce premature convergence, demonstrating improved performance in breast cancer gene selection.

For brain tumor detection, Asif et al. [39] used PSO to optimize ensemble weights in a multi-model DNN framework, achieving up to 99.66% accuracy across MRI datasets.

Collectively, these studies demonstrated that meta-heuristic, swarm-intelligence-based methods were highly effective for feature selection in medical imaging. By adaptively exploring high-dimensional search spaces, these techniques enhanced diagnostic accuracy, improved model robustness, and integrated seamlessly with classical classifiers or ensemble frameworks, offering clear advantages for complex biomedical datasets.

## 3. Problem Formulation

This study addresses the task of classifying endometrial status from conventional ultrasound images, aiming to assist clinical diagnosis of endometrial diseases characterized by substantial heterogeneity in endometrial proportions, lesion locations, and lesion sizes. The experiments are conducted on an endometrial ultrasound image dataset provided by the Department of Functional Examination at Changshu Second People’s Hospital, which includes multiple representative categories such as endometrial carcinoma, fibroids, blood clots, hyperplasia, normal endometrium, and polyps.

Let the dataset be $D={\left\{\left(x_{i},y_{i}\right)\right\}}_{i=1}^{N}$, where $x_{i}\in R^{M}$ denotes the grayscale and normalized ultrasound image vector, and $y_{i}\in \left\{0,1,\ldots ,C-1\right\}$ is the class label. We first obtain a nonlinear reduced representation via KPCA, retaining p=1000 components, and denote the reduced feature matrix by $X^{\left(\mathrm{KPCA}\right)}\in R^{N\times p}$.

We seek a binary selector $b\in {\left\{0,1\right\}}^{p}$ with index set $S\left(b\right)=\left\{j|b_{j}=1\right\}$ to maximize macro-F1 under an SVM with stratified K fold CV (K fold=3):

(1) $$b^{*}=\arg\max_{b\in {\left\{0,1\right\}}^{p}}\;\frac{1}{K\;fold}\sum_{K\;fold=1}^{K\;fold}F1_{\mathrm{macro}}^{\left(K\;fold\right)}\;\left(\mathrm{SVM}\left(X_{:,S(b)}^{\left(\mathrm{KPCA}\right)},\;Y\right)\right),\;s.t.\;{∥b∥}_{0}\geq 2.$$

The wrapper search is carried out by the Whale Migration Algorithm.

## 4. Methods

**Figure 1:**
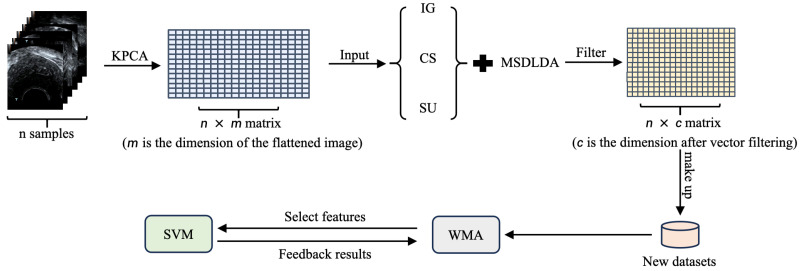
The framework of HFSOF.

Figure 1 illustrates the overall framework of the proposed HFSOF method.

### 4.1. HFSOF Phase 1: Kernel Principal Component Analysis for Nonlinear Reduction

Ultrasound texture and grayscale distributions exhibit notable nonlinearity and non-Gaussian behavior; hence, linear PCA may fail to preserve class-separating structures. KPCA leverages the kernel trick to perform linear decomposition in an implicit high-dimensional feature space, yielding nonlinear low-dimensional embeddings in the original space.

Given standardized input data $X={\left[x_{1},\ldots ,x_{N}\right]}^{⊤}\in R^{N\times d}$, where *N* denotes the number of samples and *d* the original feature dimension, a radial basis function (RBF) kernel is adopted:

(2) $$k\left(x_{i},x_{j}\right)=\exp\;\left(-\gamma ∥x_{i}-x_{j}{∥}_{2}^{2}\right),\;\gamma =\frac{1}{d}.$$

The kernel matrix $K\in R^{N\times N}$ is constructed as $K_{ij}=k\left(x_{i},x_{j}\right)$ and centered using the centering matrix $H=I_{N}-\frac{1}{N}11^{⊤}$:

(3) $$\tilde{K}=HKH.$$

Eigenvalue decomposition is then performed as $\tilde{K}=U\Lambda U^{⊤}$, where $\Lambda =\mathrm{diag}(\lambda_{1},\ldots ,\lambda_{N})$ contains eigenvalues sorted in descending order and only positive eigenvalues are retained. The projection onto kernel principal components is given by the following:

(4) $$Z=\tilde{K}\;U\;\Lambda^{-\frac{1}{2}}\in R^{N\times p}.$$

In this study, the number of retained components was set to p=1000 to balance information preservation and computational efficiency. The resulting nonlinear embedding is denoted as $X^{\left(\mathrm{KPCA}\right)}\equiv Z\in R^{N\times 1000}$ and serves as input for subsequent feature selection and optimization.

### 4.2. HFSOF Phase 2-1: Ensemble Filter Feature Selection with Median Fusion

Although KPCA reduces dimensionality, the transformed feature space may still contain redundant or weakly informative features. To further enhance robustness and mitigate overfitting, a filter-based feature selection strategy is applied. Filter methods are classifier-independent and evaluate feature relevance through statistical criteria, enabling efficient identification of discriminative features.

For each feature *j* in $X^{\left(\mathrm{KPCA}\right)}$, three filter scores are computed and subsequently fused via median-based rank aggregation to obtain a robust unified ordering.

#### 4.2.1. Information Gain (IG)

Information Gain is approximated by mutual information between the feature and the class label:

(5) $${\mathrm{IG}}_{j}=I\;\left(X_{j}^{\left(\mathrm{KPCA}\right)};Y\right)=\sum_{x,y}p\left(x,y\right)\log\frac{p(x,y)}{p(x)p(y)},$$

where Y∈{1,…,C} denotes the class label and *C* is the total number of classes.

#### 4.2.2. Chi-Square (CS)

After nonnegative transformation and discretization, the chi-square statistic is computed from the contingency table with observed counts $O_{a,c}$ and expected counts $E_{a,c}$:

(6) $$\chi_{j}^{2}=\sum_{a}\sum_{c=1}^{C}\frac{{\left(O_{a,c}-E_{a,c}\right)}^{2}}{E_{a,c}},$$

quantifying the dependence between feature *j* and the class distribution.

#### 4.2.3. Symmetrical Uncertainty (SU)

Symmetrical Uncertainty is a normalized mutual information measure:

(7) $${\mathrm{SU}}_{j}=\frac{2\;I\;\left(X_{j}^{\left(\mathrm{KPCA}\right)};Y\right)}{H\;\left(X_{j}^{\left(\mathrm{KPCA}\right)}\right)+H\left(Y\right)},\;{\mathrm{SU}}_{j}\in \left[0,1\right],$$

where H(·) denotes Shannon entropy.

#### 4.2.4. Median Fusion

Let $r_{j}^{\mathrm{IG}}$, $r_{j}^{\mathrm{CS}}$, and $r_{j}^{\mathrm{SU}}$ denote the ranks induced by IG, CS, and SU, respectively (smaller ranks indicate higher relevance). The fused rank is defined as follows:

(8) $$r_{j}^{\mathrm{fusion}}=\mathrm{median}\left\{r_{j}^{\mathrm{IG}},\;r_{j}^{\mathrm{CS}},\;r_{j}^{\mathrm{SU}}\right\}.$$

### 4.3. HFSOF Phase 2-2: Maximum Scatter Difference LDA Thresholding

Despite filter-based fusion, residual redundancy and noise may persist, particularly under small-sample and high-dimensional conditions common in endometrial ultrasound imaging. To further refine the feature set, MSDLDA is employed. Unlike classical Fisher LDA, which maximizes a scatter ratio, MSDLDA uses the difference between between-class and within-class scatter, thereby avoiding matrix inversion and alleviating singularity issues.

For feature *j*, the global mean is as follows:

(9) $$\mu_{j}=\frac{1}{N}\sum_{i=1}^{N}x_{i,j}^{\left(\mathrm{KPCA}\right)}.$$

For class c∈{1,…,C} with $n_{c}$ samples, the class mean is $\mu_{j,c}$. The between-class and within-class scatters are defined as follows:

(10) $$\begin{array}{cc} S_{B}\left(j\right) & =\sum_{c=1}^{C}n_{c}{\left(\mu_{j,c}-\mu_{j}\right)}^{2}, \end{array}$$

(11) $$\begin{array}{cc} S_{W}\left(j\right) & =\sum_{c=1}^{C}\sum_{i:\;y_{i}=c}{\left(x_{i,j}^{\left(\mathrm{KPCA}\right)}-\mu_{j,c}\right)}^{2}. \end{array}$$

The MSDLDA score is then

(12) $${\mathrm{MS}}_{j}=S_{B}\left(j\right)-S_{W}\left(j\right),$$

where larger values indicate stronger discriminative capability. Given a significance level α, let $\tau_{\alpha }$ denote the (1−α) quantile of $\left\{{\mathrm{MS}}_{j}\right\}$. Features satisfying

(13) $$S_{0}=\left\{\;j|{\mathrm{MS}}_{j}\geq \tau_{\alpha }\;\right\}$$

are retained for wrapper optimization.

### 4.4. HFSOF Phase 3: WMA for Wrapper Optimization

Although $S_{0}$ represents a refined feature subset, inter-feature interactions may still influence classification. Therefore, a wrapper-based optimization strategy is adopted, directly evaluating candidate subsets using an SVM classifier with macro-averaged F1-score as the fitness function.

#### 4.4.1. Encoding and Binarization

Each individual is encoded as a continuous vector $p\in {\left[0,1\right]}^{d}$, where $d=|S_{0}|$. A binary selection vector is obtained via

(14) $$b=I\;\left(p>\frac{1}{2}\right)\in {\left\{0,1\right\}}^{d},$$

with ${∥b∥}_{0}\geq 2$ enforced to avoid degenerate solutions.

#### 4.4.2. Fitness

An RBF-kernel SVM with fixed hyperparameters (C=100, γ=0.01) is used. The fitness function is defined as

(15) $$J\left(b\right)=\frac{1}{K\;fold}\sum_{k=1}^{K\;fold}F1_{\mathrm{macro}}^{\left(K\;fold\right)},\;K\;fold=3,$$

where K fold denotes the number of cross-validation folds. The macro-F1 score is

(16) $$F1_{\mathrm{macro}}=\frac{1}{C}\sum_{c=1}^{C}\frac{2\;P_{c}\;R_{c}}{P_{c}+R_{c}},$$

with

(17) $$P_{c}=\frac{{\mathrm{TP}}_{c}}{{\mathrm{TP}}_{c}+{\mathrm{FP}}_{c}},\;R_{c}=\frac{{\mathrm{TP}}_{c}}{{\mathrm{TP}}_{c}+{\mathrm{FN}}_{c}}.$$

#### 4.4.3. Leaders and Followers Update

With population size $n_{\mathrm{pop}}=45$ and maximum iterations T=100, the top $\left\lceil n_{\mathrm{pop}}/2\right\rceil$ individuals are designated as leaders. Let $m^{\left(t\right)}$ denote the mean position of leaders and $p^{*(t)}$ the best individual at iteration *t*. Updates are performed as

(18) $$\begin{array}{cc} \mathrm{Leader}:\; & p^{\left(t+1\right)}=\mathrm{clip}\;\left(p^{\left(t\right)}+u⊙v,\;0,1\right), \end{array}$$

(19) $$\begin{array}{cc} \mathrm{Follower}:\; & p^{\left(t+1\right)}=\mathrm{clip}\;\left(m^{\left(t\right)}+r_{1}⊙\;\left(p_{\mathrm{prev}}^{\left(t\right)}-p^{\left(t\right)}\right)+r_{2}⊙\;\left(p^{*(t)}-m^{\left(t\right)}\right),\;0,1\right), \end{array}$$

where $u∼U{\left(-1,1\right)}^{d}$, $v,r_{1},r_{2}∼U{\left(0,1\right)}^{d}$, and ⊙ denotes the Hadamard product. Greedy selection retains the better solution between old and updated individuals. The globally best solution $b^{*}$ yields the final feature subset $S^{*}=S\left(b^{*}\right)$.

## 5. Experiments

### 5.1. Dataset and Experimental Preparation

This study conducted experiments using the endometrial conventional ultrasound image dataset provided by the Department of Functional Examination at Changshu Second People’s Hospital. The dataset contains a total of 7680 endometrial images, including 1183 images of endometrial carcinoma, 660 images of uterine fibroids, 238 images of endometrial blood clots, 1392 images of endometrial hyperplasia, 743 normal images, and 3464 images of endometrial polyps. These images exhibit significant inter-group differences in endometrial proportion, lesion location, and lesion size, reflecting the clinical heterogeneity of endometrial diseases.

#### 5.1.1. Preprocessing and Partitioning

All images are converted to grayscale, normalized to [0,1], flattened, and then standardized (zero mean, unit variance). A stratified train/test split of 70%/30% is adopted to preserve class proportions.

#### 5.1.2. Experimental Settings

The WMA was configured with a population size of 45 and run for 100 iterations. The selected feature subsets were evaluated using a Support Vector Machine with an RBF kernel, where the regularization parameter was set to C=100 and the kernel width to γ=0.01.

#### 5.1.3. Clinical Rationale, Imaging Protocol, and Data Annotation

Differentiating endometrial conditions is clinically important, as different lesion types exhibit distinct contrast-enhanced ultrasound (CEUS) patterns and imply different pathological behaviors, treatment strategies, and prognoses. In this study, six categories of endometrial conditions were considered, each characterized by specific CEUS features. Endometrial carcinoma (EC) typically presents early enhancement, high enhancement intensity, and early wash-out, often accompanied by neovascularization and a tendency to invade the myometrium. Uterine fibroids commonly show ring-like enhancement patterns, and in some cases may exhibit appearances similar to endometrial polyps. Endometrial polyps often exhibit papillary, droplet-like, tongue-shaped, or cord-like root structures, with punctate or strip-like signals of penetrating blood flow. Endometrial hyperplasia is characterized by uniform endometrial thickening with homogeneous or heterogeneous but generally increased echogenicity. Blood clots or purulent inflammation typically appear as distinct low-contrast “black hole” regions in which the contrast agent fails to penetrate.

All ultrasound images were acquired using a Mindray Resona 7S color Doppler ultrasound diagnostic system (Shenzhen Mindray Bio-Medical Electronics Co., Ltd., Shenzhen, China), which supports real-time CEUS matching imaging and time–intensity curve analysis. Examinations were performed using a V11-3HC endocavitary transducer with a frequency range of 3.0–11.0 MHz. The mechanical index was maintained between 0.08 and 0.10 to ensure low-acoustic-pressure imaging suitable for contrast-enhanced studies. Imaging protocols were kept consistent across patients to minimize acquisition variability.

Image selection and labeling were based on pathological examination, which served as the clinical gold standard. Pathological confirmation was obtained for patients with suspected lesions, while cases without suspected abnormalities did not undergo invasive pathological assessment. This strategy reflects routine clinical practice and ensures that the reference labels used in this study are clinically reliable.

#### 5.1.4. Computing Environment Specifications

All experiments were conducted on a high-performance computing server equipped with dual Intel® Xeon® Gold 6338 CPUs (2.00 GHz; Intel Corporation, Santa Clara, CA, USA). Each processor provides 32 physical cores with hyper-threading enabled. In our experiments, a total of 56 physical CPU cores were utilized for model training and feature selection to ensure efficient computation.

### 5.2. Evaluation Metrics

We evaluate performance at both per-class and aggregated levels using Accuracy, Precision, Recall, F1 score, and multi-class one-vs-rest (OVR) AUC.

#### 5.2.1. Notation

Let the number of classes be *C* and the total number of samples be *N*. For each class c∈{1,…,C}, denote

$${\mathrm{TP}}_{c},\;{\mathrm{FP}}_{c},\;{\mathrm{FN}}_{c},\;{\mathrm{TN}}_{c}$$

as true positives, false positives, false negatives, and true negatives, respectively. Let $n_{c}$ be the support (number of samples) of class *c*, so $\sum_{c=1}^{C}n_{c}=N$.

#### 5.2.2. Accuracy and Per-Class Metrics

Accuracy:

(20) $$\mathrm{Acc}=\frac{1}{N}\sum_{c=1}^{C}{\mathrm{TP}}_{c}.$$

Class-wise Precision and Recall:

(21) $$\begin{array}{cc} P_{c} & =\frac{{\mathrm{TP}}_{c}}{{\mathrm{TP}}_{c}+{\mathrm{FP}}_{c}}, \end{array}$$

(22) $$\begin{array}{cc} R_{c} & =\frac{{\mathrm{TP}}_{c}}{{\mathrm{TP}}_{c}+{\mathrm{FN}}_{c}}. \end{array}$$

Class-wise F1 score:

(23) $$F1_{c}=\frac{2\;P_{c}\;R_{c}}{P_{c}+R_{c}}.$$

#### 5.2.3. Macro vs. Weighted Averages

Macro-averaged: equal weight to each class, emphasizing class-level balance:

(24) $$\begin{array}{cc} {\mathrm{Precision}}_{\mathrm{macro}} & =\frac{1}{C}\sum_{c=1}^{C}P_{c}, \end{array}$$

(25) $$\begin{array}{cc} {\mathrm{Recall}}_{\mathrm{macro}} & =\frac{1}{C}\sum_{c=1}^{C}R_{c}, \end{array}$$

(26) $$\begin{array}{cc} F1_{\mathrm{macro}} & =\frac{1}{C}\sum_{c=1}^{C}F1_{c}. \end{array}$$

Weighted-averaged: weighted by class support $n_{c}$, reflecting label imbalance:

(27) $$\begin{array}{cc} {\mathrm{Precision}}_{\mathrm{weighted}} & =\frac{1}{N}\sum_{c=1}^{C}n_{c}\;P_{c}, \end{array}$$

(28) $$\begin{array}{cc} {\mathrm{Recall}}_{\mathrm{weighted}} & =\frac{1}{N}\sum_{c=1}^{C}n_{c}\;R_{c}, \end{array}$$

(29) $$\begin{array}{cc} F1_{\mathrm{weighted}} & =\frac{1}{N}\sum_{c=1}^{C}n_{c}\;F1_{c}. \end{array}$$

because $R_{c}={\mathrm{TP}}_{c}/n_{c}$ and $\sum_{c}n_{c}R_{c}=\sum_{c}{\mathrm{TP}}_{c}$.

#### 5.2.4. AUC

For multi-class problems, we adopt one-vs-rest: treat class *c* as positive and all others as negative to compute a per-class ROC curve and its AUC, denoted ${\mathrm{AUC}}_{c}$:

(30) $$\begin{array}{cc} {\mathrm{AUC}}_{\mathrm{macro}} & =\frac{1}{C}\sum_{c=1}^{C}{\mathrm{AUC}}_{c}, \end{array}$$

(31) $$\begin{array}{cc} {\mathrm{AUC}}_{\mathrm{weighted}} & =\frac{1}{N}\sum_{c=1}^{C}n_{c}\;{\mathrm{AUC}}_{c}. \end{array}$$

Macro AUC emphasizes equal per-class separability, while weighted AUC mirrors the label distribution. Under class imbalance, reporting both provides a balanced view of performance on minority and majority classes.

### 5.3. Experimental Results Table

#### 5.3.1. HFSOF Method Effect Table

The table below summarizes the performance results across the three phases of the HFSOF framework. In Phase 1, features extracted by KPCA were directly used as input to an SVM classifier. Phase 2 employed the integrated three-filter approach combined with MSDLDA for feature selection, followed by classification with SVM. In Phase 3, the Whale Migration Algorithm was applied for optimized feature selection prior to SVM classification, yielding the final performance results.

Table 1 and Table 2 present the macro-averaged and weighted-averaged performance metrics of the HFSOF framework across the three phases.

From Table 1, it is evident that Phase 1, which directly applies KPCA features to the SVM classifier, achieves a high macro precision of 93.15%, indicating that the model correctly identifies positive samples with high accuracy. However, the macro recall is only 49.71%, suggesting that many relevant instances are missed, leading to a relatively low F1 score of 0.5927. Phase 2 demonstrates a significant improvement in recall (69.27%) and F1 score (0.7763) due to the integration of the three-filter feature selection approach combined with MSDLDA, while maintaining high precision. Phase 3, which incorporates the Whale Migration Algorithm for optimized feature selection, achieves the best overall performance with macro precision of 94.52%, macro recall of 76.05%, and macro F1 of 0.8244, indicating balanced and robust classification performance. The macro AUC remains consistently high across all phases, showing that the model has strong discriminative capability.

Table 2 reports the weighted-averaged metrics, which consider the class distribution. The weighted precision and recall increase progressively from Phase 1 to Phase 3, reflecting the effectiveness of the feature selection and optimization strategies in handling class imbalance. Specifically, Phase 3 achieves the highest weighted F1 score of 0.859, highlighting its superior overall performance. The weighted AUC remains above 0.97 in all phases, indicating reliable predictive performance across classes.

Overall, the experimental results confirm that each stage of the HFSOF framework contributes to improved classification performance, with Phase 3 providing the most balanced and accurate predictions.

#### 5.3.2. Comparative Experiments of HFSOF Phase 2-1 (Replace the Filter)

The two tables below present the experimental results obtained with a single filter and the MSDLDA method.

Table 3 and Table 4 present an ablation study comparing the integrated three-filter approach combined with MSDLDA (Phase 2) against the use of individual filters (IG, Chi-square, SU) combined with MSDLDA, with significance level α=0.05.

From Table 3, the Phase 2 integrated approach achieves a macro precision of 94.20%, macro recall of 69.27%, macro F1 score of 0.7763, and macro AUC of 0.9789. Although some single-filter methods achieve slightly higher recall and F1 values, their macro precision and macro AUC are consistently lower than Phase 2. This indicates that the integrated three-filter approach provides a better balance between precision and recall, resulting in more robust overall classification performance.

Similarly, Table 4 shows that Phase 2 outperforms single-filter methods on weighted-averaged metrics that account for class distribution. Phase 2 achieves weighted precision of 86.56%, weighted recall of 82.20%, weighted F1 of 0.8173, and weighted AUC of 0.9811. The single-filter methods, while competitive in weighted recall and F1, do not match Phase 2 in precision and AUC, suggesting that the integrated filtering strategy better preserves discriminative features across all classes.

Overall, these results confirm that the Phase 2 method, combining multiple filters with MSDLDA, is highly effective. By leveraging the complementary strengths of the individual filters, it provides a more stable and balanced feature selection, leading to superior classification performance compared to using any single filter alone.

#### 5.3.3. Comparative Experiments of HFSOF Phase 2-1 (Replace the Simple Average Fusion)

The two tables below show the effect of replacing the original median fusion method in Phase 2 with simple average fusion.

Table 5 and Table 6 compare the performance of the Phase 2 method using the original median fusion strategy with an alternative simple average fusion approach.

As shown in Table 5, the median-fused Phase 2 method achieves a macro precision of 94.20%, macro recall of 69.27%, macro F1 of 0.7763, and macro AUC of 0.9789. In contrast, using simple average fusion results in lower macro precision (86.12%) and macro AUC (0.9549), although the recall and F1 are comparable. This indicates that median fusion more effectively preserves the most discriminative features from the three-filter integration, yielding higher precision and improved overall class separability.

Similarly, Table 6 shows that median fusion consistently outperforms simple average fusion in weighted-averaged metrics. Phase 2 with median fusion achieves weighted precision of 86.56%, weighted recall of 82.20%, weighted F1 of 0.8173, and weighted AUC of 0.9811, surpassing the simple average fusion variant in both precision and AUC. These results highlight that the median fusion strategy provides a more robust and balanced representation of feature importance, contributing to the superior performance of Phase 2.

Overall, the results confirm that the use of median fusion in Phase 2 is a key factor in achieving stable and accurate classification performance across all evaluation metrics.

#### 5.3.4. Comparative Experiments of HFSOF Phase 2-2 (Replace the α)

The two tables below show the changes in the evaluation metrics when the α value of the MSDLDA method is changed.

Table 7 and Table 8 present the experimental results of Phase 2 when varying the significance level α in the MSDLDA method.

For the macro-averaged metrics shown in Table 7, Phase 2 with α=0.05 achieves a macro precision of 94.20%, macro recall of 69.27%, macro F1 of 0.7763, and macro AUC of 0.9789. When α is decreased to 0.01, the macro precision slightly decreases to 90.54%, while recall and F1 improve marginally. However, the macro AUC decreases to 0.9668, indicating a slight reduction in overall discriminative ability. Increasing α to 0.1 significantly degrades all metrics, with macro precision dropping to 51.89% and macro F1 to 0.4960, reflecting that an overly lenient significance threshold allows many less relevant features, negatively impacting classification performance.

Similarly, the weighted-averaged metrics in Table 8 show that Phase 2 with α=0.05 provides a balanced and robust performance across all classes, with weighted precision of 86.56%, weighted recall of 82.20%, weighted F1 of 0.8173, and weighted AUC of 0.9811. Using α=0.01 slightly increases weighted recall and F1 but reduces precision and AUC, while α=0.1 leads to severe performance degradation.

The significance level α directly controls the pruning strength in MSDLDA and thus affects the size of the initial feature set $S_{0}$. Smaller values of α enforce stricter statistical significance criteria, leading to more aggressive feature elimination and a more compact $S_{0}$. In contrast, larger α values relax the pruning condition, allowing more features to be retained. The experimental results in Table 7 and Table 8 confirm this monotonic relationship and illustrate the trade-off between feature compactness and information preservation.

These results demonstrate that the choice of α=0.05 in Phase 2 achieves an optimal trade-off between selecting statistically significant features and maintaining discriminative power, resulting in the most stable and accurate classification performance.

#### 5.3.5. Comparative Experiments of HFSOF Phase 3

The two tables below show the effect of replacing the WMA algorithm with the PSO algorithm.

Table 9 and Table 10 compare the performance of the HFSOF framework in Phase 3 when using the WMA versus the PSO algorithm for feature selection.

As shown in Table 9, the WMA-based approach achieves a macro precision of 94.52%, macro recall of 76.05%, macro F1 of 0.8244, and macro AUC of 0.9793. The PSO-based variant has slightly lower macro precision (91.56%) and macro AUC (0.9735), while its recall is comparable. These results indicate that WMA more effectively selects a subset of features that enhances overall class separability and maintains high precision across all classes.

Similarly, Table 10 demonstrates that WMA outperforms PSO in weighted-averaged metrics as well. The WMA-based method achieves weighted precision of 87.99%, weighted recall of 86.20%, weighted F1 of 0.859, and weighted AUC of 0.9779, surpassing the PSO-based method in all metrics. This highlights the stability and robustness of WMA in optimizing the feature subset for imbalanced and multi-class datasets.

Overall, these results confirm that the WMA-based feature selection in Phase 3 provides the most effective and balanced classification performance, making it the preferred optimization strategy within the HFSOF framework.

#### 5.3.6. Classification Performance of the Proposed HFSOF Method

**Figure 2:**
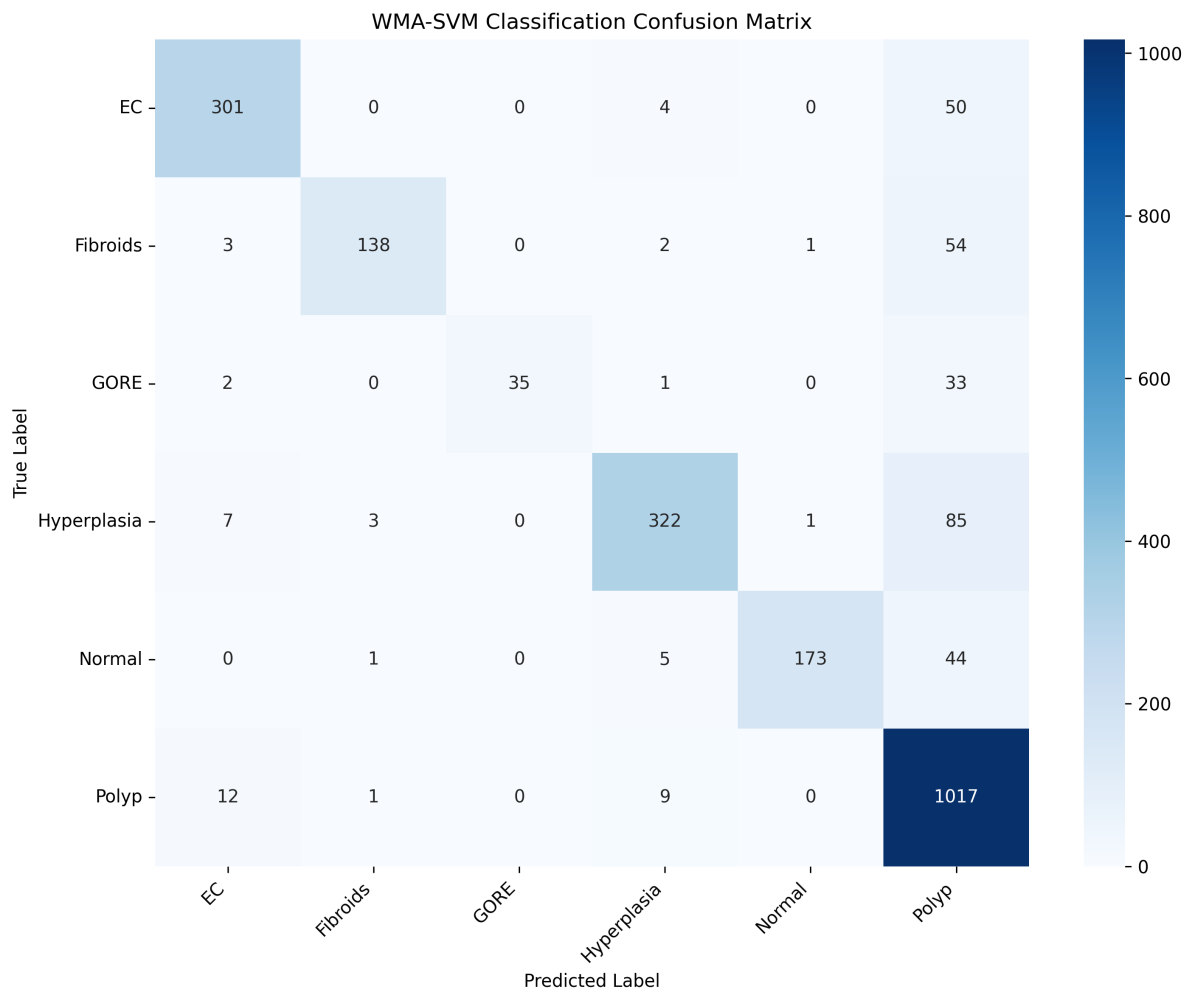
Confusion matrix of the proposed HFSOF method on the endometrial ultrasound dataset.

To further evaluate the classification performance of the proposed HFSOF method, we additionally present the confusion matrix, as shown in Figure 2, together with detailed class-wise performance metrics.

As shown in Table 11, the proposed HFSOF method achieves consistently high precision across most categories, indicating a low false-positive rate in endometrial disease classification. In particular, EC, Fibroids, GORE, Hyperplasia, and Normal classes all obtain precision values above 93%, demonstrating the strong discriminative capability of the proposed method.

In terms of recall, the Polyp class achieves the highest recall (98.00%), suggesting that HFSOF is highly effective in identifying polyp lesions, which is partly attributed to the relatively larger number of samples in this category. By contrast, GORE and Fibroids exhibit comparatively lower recall values, reflecting the increased difficulty in correctly identifying these classes due to their limited sample size and visual similarity to other endometrial abnormalities.

Overall, the F1-score results indicate a favorable balance between precision and recall for most categories, with EC achieving an F1-score of 89.00%, which is clinically significant given the importance of accurate endometrial carcinoma detection. These results demonstrate that the proposed HFSOF method is robust to inter-class variability and capable of handling the clinical heterogeneity present in endometrial ultrasound images. However, the classification performance for the GORE category is relatively limited, which may be attributed to its small sample size and visual similarity to other endometrial abnormalities. In future work, incorporating deep learning-based feature representations or end-to-end learning frameworks may further enhance discriminative performance for challenging categories such as GORE.

#### 5.3.7. Discriminative Feature Subspace Visualization of the Proposed HFSOF Method

To further investigate the discriminative capability of the feature subset selected by the proposed HFSOF method, a two-dimensional visualization is conducted using Uniform Manifold Approximation and Projection (UMAP). The UMAP projection is applied to the weighted multi-feature representation after feature selection, enabling an intuitive assessment of the intrinsic structure and class separability in the learned feature subspace.

**Figure 3:**
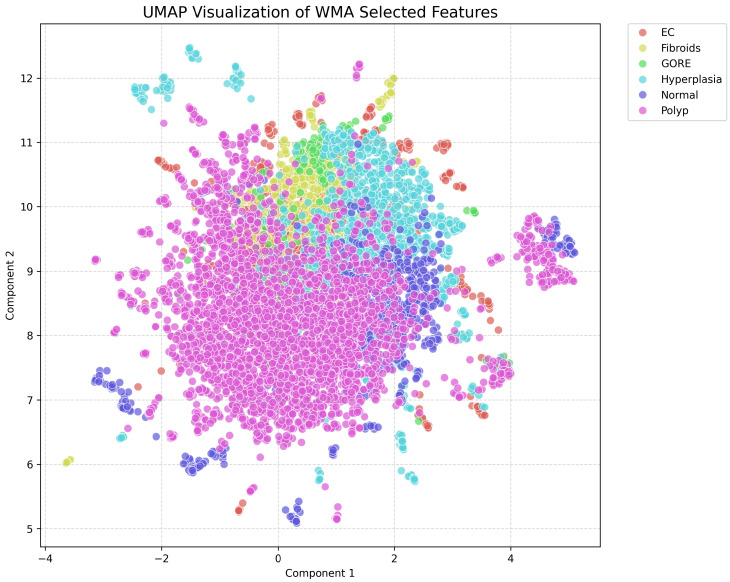
UMAP visualization of the feature subspace selected by the proposed HFSOF method on the endometrial ultrasound dataset.

As illustrated in Figure 3, samples from different categories exhibit clear clustering tendencies while preserving reasonable inter-class boundaries. The Normal and Hyperplasia classes form relatively compact and coherent clusters, indicating stable intra-class feature distributions. In contrast, EC and GORE samples are distributed in adjacent but distinguishable regions, reflecting their pathological similarity while still maintaining discriminative margins in the selected feature space.

Fibroid and Polyp samples exhibit moderate overlap with surrounding categories, consistent with their heterogeneous imaging appearances on contrast-enhanced ultrasound. Nevertheless, these classes still present identifiable density centers, suggesting that the proposed HFSOF method effectively retains class-relevant information while suppressing redundant or noisy features.

Overall, the UMAP visualization confirms that the feature subset selected by HFSOF yields a structured, discriminative feature subspace, facilitating downstream classification tasks. This observation is well aligned with the quantitative classification results, indicating that the proposed method enhances both feature interpretability and classification robustness.

### 5.4. Optimization Time Comparison

To further quantify the computational cost of the two wrapper optimizers, we compare the optimization time for WMA and PSO.

Table 12 compares the computational cost of the two wrapper-based optimizers in Phase 3. Here, the optimization time refers to the total time required for population updates and decoding of candidate feature subsets.

The WMA-based optimizer completes the feature selection process in 4095.23 s, while the PSO-based optimizer requires 4220.65 s. This indicates that WMA not only provides superior classification performance, as shown in Table 9 and Table 10, but also achieves faster convergence and lower computational cost.

It should be noted that this computational cost mainly corresponds to the offline training and optimization stage of the WMA framework. Once the optimal feature subset is determined and the classifier is fully trained, the deployment stage is highly efficient: the inference time for a single unseen sample is approximately 2 s, which is fully acceptable for practical medical applications.

Overall, WMA demonstrates both effectiveness in selecting high-quality feature subsets and efficiency in runtime, making it a favorable choice for Phase 3 optimization within the HFSOF framework.

## 6. Conclusions

This study proposes an HFSOF framework to address the challenges of diagnosing endometrial lesions using ultrasound images, implementing a closed-loop process that spans nonlinear dimensionality reduction, feature fusion and selection, and intelligent optimization and classification. Experimental results demonstrate that KPCA effectively preserves the nonlinear structures of ultrasound images, the integrated three-filter strategy combined with MSDLDA enables robust and discriminative feature selection, and the Whale Migration Algorithm further optimizes the feature subset under the SVM classifier constraint. As a result, the proposed framework achieves significant improvements in macro-averaged F1 score and AUC, exhibiting comprehensive advantages in precision, recall, and class balance.

Nevertheless, several limitations remain. Despite the overall strong performance of HFSOF, the imbalanced nature of the endometrial lesion dataset may constrain recall for minority classes, potentially affecting the comprehensiveness and reliability of clinical diagnosis. In addition, although the framework is designed to be device-agnostic, its performance may still be influenced by variations across ultrasound devices and imaging protocols. Differences in image reconstruction algorithms, probe characteristics, operating frequencies, and acquisition settings may lead to domain shifts in texture patterns and temporal dynamics that are not fully captured by the current single-center dataset. Moreover, the current method has been validated on medium-scale datasets, and its generalization to large-scale, multi-center, or heterogeneous ultrasound datasets requires further systematic evaluation.

Future research may focus on the following directions: first, incorporating imbalance-handling strategies (e.g., class re-sampling, cost-sensitive learning, or adversarial sample augmentation) to improve minority class recognition; second, extending the framework to multi-center and multi-device datasets and introducing domain adaptation or feature normalization strategies to enhance robustness across different ultrasound platforms and acquisition protocols; third, exploring multimodal image fusion and transferable feature representations to further improve generalizability. Finally, integrating HFSOF with interpretable or explainable models may provide quantifiable and clinically meaningful decision support, thereby facilitating its translation into real-world AI-assisted ultrasound diagnosis.

## Figures and Tables

**Table 1:** Macro-averaged metrics.

Phase	Macro Precision	Macro Recall	Macro F1	Macro AUC
Phase 1	93.15%	49.71%	0.5927	0.9968
Phase 2	94.20%	69.27%	0.7763	0.9789
**Phase 3**	**94.52%**	**76.05%**	**0.8244**	**0.9793**

**Table 2:** Weighted-averaged metrics.

Phase	Weighted Precision	Weighted Recall	Weighted F1	Weighted AUC
Phase 1	81.47%	68.53%	0.6611	0.9985
Phase 2	86.56%	82.20%	0.8173	0.9811
**Phase 3**	**87.99%**	**86.20%**	**0.859**	**0.9779**

**Table 3:** Macro-averaged metrics for integrated and single filter methods combined with MSDLDA (α=0.05).

Experiment	Macro Precision	Macro Recall	Macro F1	Macro AUC
**Phase 2**	**94.20%**	**69.27%**	**0.7763**	**0.9789**
Only IG	91.15%	73.15%	0.7973	0.9700
Only Chi-square	92.74%	72.63%	0.7985	0.9743
Only SU	91.15%	73.15%	0.7973	0.9700

**Table 4:** Weighted-averaged metrics for integrated and single filter methods combined with MSDLDA (α=0.05).

Experiment	Weighted Precision	Weighted Recall	Weighted F1	Weighted AUC
**Phase 2**	**86.56%**	**82.20%**	**0.8173**	**0.9811**
Only IG	85.58%	83.64%	0.8332	0.9713
Only Chi-square	86.09%	83.46%	0.8312	0.9759
Only SU	85.58%	83.64%	0.8332	0.9713

**Table 5:** Macro-averaged metrics comparing median-fused with simple average fusion.

Experiment	Macro Precision	Macro Recall	Macro F1	Macro AUC
**Phase 2**	**94.20%**	**69.27%**	**0.7763**	**0.9789**
Simple average fusion	86.12%	72.60%	0.7775	0.9549

**Table 6:** Weighted-averaged metrics comparing median-fused with simple average fusion.

Experiment	Weighted Precision	Weighted Recall	Weighted F1	Weighted AUC
**Phase 2**	**86.56%**	**82.20%**	**0.8173**	**0.9811**
Simple average fusion	82.61%	81.81%	0.8145	0.9569

**Table 7:** Macro-averaged metrics under different MSDLDA significance levels (α).

Experiment	Macro Precision	Macro Recall	Macro F1	Macro AUC
**Phase 2 (α=0.05)**	**94.20%**	**69.27%**	**0.7763**	**0.9789**
MSDLDA (α=0.01)	90.54%	72.79%	0.7917	0.9668
MSDLDA (α=0.1)	51.89%	48.00%	0.4960	0.8333

**Table 8:** Weighted-averaged metrics under different MSDLDA significance levels (α).

Experiment	Weighted Precision	Weighted Recall	Weighted F1	Weighted AUC
**Phase 2 (α=0.05)**	**86.56%**	**82.20%**	**0.8173**	**0.9811**
MSDLDA (α=0.01)	85.18%	83.46%	0.8308	0.9693
MSDLDA (α=0.1)	56.39%	57.12%	0.5634	0.8076

**Table 9:** Macro-averaged metrics comparing WMA and PSO optimizers.

Experiment	Macro Precision	Macro Recall	Macro F1	Macro AUC
**WMA-based**	**94.52%**	**76.05%**	**0.8244**	**0.9793**
PSO-based	91.56%	76.29%	0.8217	0.9735

**Table 10:** Weighted-averaged metrics comparing WMA and PSO optimizers.

Experiment	Weighted Precision	Weighted Recall	Weighted F1	Weighted AUC
**WMA-based**	**87.99%**	**86.20%**	**0.859**	**0.9779**
PSO-based	86.95%	85.46%	0.8518	0.9752

**Table 11:** Detailed class-wise classification performance on the endometrial ultrasound dataset obtained using the proposed HFSOF method.

Class	Precision	Recall	F1-Score	Support
EC	93.00%	85.00%	89.00%	355
Fibroids	97.00%	70.00%	81.00%	198
GORE	100.00%	49.00%	66.00%	71
Hyperplasia	94.00%	77.00%	85.00%	418
Normal	99.00%	78.00%	87.00%	223
Polyp	79.00%	98.00%	88.00%	1039

**Table 12:** Runtime comparison of two optimizers.

Swarm Intelligence Algorithm Name	Optimization Time (s)
**WMA-based**	**4095.2339**
PSO-based	4220.6541

## Data Availability

The original contributions presented in this study are included in the article. Further inquiries can be directed to the corresponding authors.
